# From Maternal Exposure to F1 Development: Unveiling Cyclophosphamide-Induced Reproductive Toxicity

**DOI:** 10.3390/biomedicines14061353

**Published:** 2026-06-16

**Authors:** Xiaolin Meng, Fengyuan Liu, Na Xu, Jihui Ai, Jie Yang, Hualin Bai, Qiuyue Liao, Yan Zhang, Jianliu Wang, Jianbo Wei, Kezhen Li

**Affiliations:** 1 Department of Obstetrics and Gynecology, Tongji Hospital, Tongji Medical College, Huazhong University of Science and Technology, Wuhan 430030, China; mengxiaolin01@163.com (X.M.);; 2 National Clinical Research Center for Obstetrics and Gynecology, Cancer Biology Research Center, Key Laboratory of the Ministry of Education, Tongji Hospital, Tongji Medical College, Huazhong University of Science and Technology, Wuhan 430030, China; 3 Shanghai First Maternity and Infant Hospital, Shanghai 201204, China; 4 Department of Obstetrics and Gynecology, Peking University People’s Hospital, Beijing 100033, China; 5 Department of Obstetrics and Gynecology, First Affiliated Hospital of Guangzhou Medical University, Guangzhou 510120, China

**Keywords:** cyclophosphamide, ovarian function, fertility, offspring, developmental toxicity

## Abstract

**Background**: Various controversial conclusions exist regarding the reproductive toxicity of cyclophosphamide, creating uncertainties about the recovery timeline of maternal reproductive capacity and offspring health. **Methods**: Using a mouse model with a clinically relevant cyclophosphamide dosing regimen, we examined the recovery of female reproductive function after exposure and the long-term survival and development of their offspring. **Results**: Our findings revealed that cyclophosphamide exposure shortened the maternal reproductive lifespan, characterized by early fertility impairment at one week (*p* < 0.05), transient recovery at two weeks (*p* > 0.05), a subsequent decline at four weeks with further deterioration, and eventual progression to infertility at six months (*p* < 0.01). F1 pups from the cyclophosphamide group exhibited growth restriction, higher mortality rates, delayed pubertal onset, and impaired neurodevelopment during long-term follow-up. Although some parameters transiently improved at 2 weeks post-withdrawal, these abnormalities persisted or recurred at 4 and 8 weeks, indicating that developmental defects were not lessened by prolonging the medication withdrawal period. **Conclusions**: These findings demonstrate irreversible gonadotoxicity and developmental toxicity following cyclophosphamide exposure in this mouse model.

## 1. Introduction

With the rising incidence of malignant tumors among younger populations, survival rates among young cancer patients have also increased [1]. However, improved survival among young female cancer patients has increased concerns regarding post-treatment fertility and potential adverse effects on offspring health due to chemotherapy-induced gonadotoxicity [2,3,4,5]. A comprehensive understanding of the adverse reproductive effects following chemotherapy exposure is crucial. This involves not only assessing ovarian function and fertility potential in females, but also examining obstetric and pediatric outcomes, as well as potential long-term effects on offspring.

Cyclophosphamide (CTX) is a widely used alkylating agent and a cornerstone drug in treatment regimens for breast cancer [6], lymphoma [7,8,9,10], systemic lupus erythematosus [11], and it exhibits notable gonadal toxicity [12,13,14]. Previous studies in human survivors have indicated that CTX or other alkylating agents may reduce the likelihood of pregnancy [15,16,17,18], induce acute ovarian failure [19], prompt premature menopause [10,19], and increase the risks of preterm labor [20] and low birth weight infants [20]. However, available evidence has not demonstrated an increased risk of congenital anomalies in offspring [21,22,23,24]. In addition, these studies lacked continued follow-up evaluation with pregnancy and offspring outcomes, limiting the ability to determine whether delayed developmental injury may emerge later in life. Therefore, determining the possible adverse effects of maternal exposure to high-dose alkylating agents such as CTX on offspring requires large cohort studies with extended follow-up. However, such evidence remains limited. Consequently, the clinical consequences of CTX exposure on offspring safety remain inconclusive and may be underestimated [25]. The scarcity of available data may stem from the limited number of successful deliveries following chemotherapy and the challenges associated with long-term monitoring of offspring health. Limited clinical data and concerns regarding offspring safety may further contribute to intentional contraceptive behavior among patients. Furthermore, the widespread practice of mixing chemotherapeutic medicines in clinical settings introduces possible bias by making it more difficult to identify and comprehend each agent’s unique effects. In addition, a large portion of the information that is currently available is based on data from questionnaires [10,26], and the inclusion of all possible offspring was limited by non-responses to the surveys. When taken as a whole, these drawbacks emphasize the necessity of animal research to investigate optimal conception timing, obstetric outcomes, and the safety of offspring after CTX treatment.

Indeed, several animal studies have reported that conception after CTX exposure leads to embryo-fetal developmental toxicity [27,28,29,30], characterized by increased rates of fetal malformations, reduced fetal and placental weights, and embryo resorption. Meirow et al. [28] also demonstrated that conception in female mice following a single exposure to 75 mg/kg CTX led to a high rate of abortions and malformations, with malformation rates at least tenfold higher than in control groups. Consequently, although existing clinical reports are generally reassuring regarding pregnancy and obstetric outcomes, concerns remain about potential heritable effects on offspring in rodent studies. Similarly, animal research has also placed limited emphasis on the long-term developmental outcomes of offspring. Therefore, it is essential to systematically evaluate the long-term impact of CTX exposure on pregnancy outcomes in female mice, in order to better inform safe conception timing following chemotherapy in humans. However, no studies have systematically evaluated the long-term developmental trajectory of offspring conceived after different recovery intervals following repeated CTX exposure.

In this study, we employed multiple cycles of high-dose CTX, mimicking clinical drug administration patterns, to examine the long-term toxicity of pre-pregnancy CTX exposure on maternal mice and their offspring. For maternal assessment, we evaluated ovarian function, natural pregnancy outcomes, the quantity of oocytes retrieved after superovulation, and embryo developmental potential following in vitro fertilization (IVF). For offspring assessment, we investigated whether maternal exposure to repeated CTX courses led to birth defects and whether the surviving pups maintained long-term health. The study monitored various time points post-chemotherapy to determine whether outcomes improved or deteriorated as the interval between CTX cessation and natural mating increased. These findings may provide crucial insights from animal models to guide considerations in humans receiving common clinical dosages.

## 2. Materials and Methods

### 2.1. Animals

All experimental procedures were approved by the Ethics Committee of Tongji Hospital, Tongji Medical College, Huazhong University of Science and Technology (Ethics No. TJH-202009020). All animal experiments were conducted in accordance with institutional guidelines. C57BL/6 mice (female: 6–7 weeks old; male: 10–12 weeks old; GemPharmatech Co., Ltd, Chengdu, China) were housed in a specific pathogen-free (SPF) facility. Animals were maintained under controlled environmental conditions, including a constant temperature of 22 ± 2 °C and a 12-h light/dark cycle.

### 2.2. Experimental Design

After a one-week acclimatization period, female mice were randomly allocated to either the control or CTX-treated group. To establish the mouse model, we mimicked the clinically relevant repeated-dose regimen [6,8,14,26], with dose adjustments based on interspecies conversion factors. CTX (Endoxan; Baxter Oncology GmbH, Halle, Germany) was administered intraperitoneally at 150 mg/kg every 5 days for three cycles (days 1, 5, and 10), while control mice received an equal volume (200 μL) of sterile saline (Wuhan Huaren Tongji Pharmaceutical Co., Ltd., Wuhan, China) via intraperitoneal injection. Outcome assessments were conducted at 1, 2, 4, 8, and 12 weeks, as well as 6 months after the final CTX administration. Evaluations included ovarian reserve, fertility after natural mating, oocyte yield after superovulation, and embryonic developmental potential assessed by in vitro fertilization (IVF). Sample sizes for each experiment are described in the corresponding sections below and summarized in Supplementary Tables S1–S5.

### 2.3. Ovarian Histomorphology

Following euthanasia, ovaries were excised and fixed in 4% paraformaldehyde (Servicebio Technology Co., Ltd., Wuhan, China). The tissues were subsequently dehydrated, embedded in paraffin, and serially sectioned at 5 µm at the maximal ovarian cross-section. To minimize double counting, every fifth section was collected for hematoxylin and eosin (H&E) staining (Servicebio Technology Co., Ltd., Wuhan, China) and histological analysis. Follicles were classified according to their developmental stage by three investigators blinded to the treatment group. The criteria used for follicle classification are shown in Figure S1. For follicle counting analyses, *N* = 4–5 mice per group per time point were analyzed.

### 2.4. Fertility Assessment of Female Mice

At 1, 2, 4, 8, 12 weeks, and 6 months after cessation of the final CTX administration, natural mating was conducted to evaluate fertility. Female mice were housed with males at a ratio of 2:1 for one week. Two weeks after mating, females were palpated to determine pregnancy status. The number of pregnancies, total litter size (including live and stillbirths), live birth rate, and litter birth weight were recorded. Pregnancy rate was defined as the proportion of pregnant females among all cohabited females. During the perinatal period, maternal mice were monitored twice daily at fixed time points (morning and evening) to collect detailed records of pup births. This approach minimized the possibility of complete loss of neonatal records due to maternal cannibalism. For fertility assessment, *N* = 13–33 female mice per group were included in natural mating experiments.

### 2.5. Superovulation and In Vitro Fertilization (IVF)

Female mice were administered 10 IU Pregnant Mare Serum Gonadotropin (PMSG; Solarbio Science & Technology Co., Ltd., Beijing, China) via intraperitoneal injection between 5–6 pm, followed by an intraperitoneal injection of 10 IU Human Chorionic Gonadotropin (hCG; Fengyuan Pharmaceutical Co., Ltd., Maanshan, China) 48 h later. Fertilization procedures were initiated 16 h after hCG administration. On the day of fertilization, sperm were collected from the epididymal tail and vas deferens of adult male mice and incubated in prewarmed GIVF-PLUS medium (Vitrolife, Gothenburg, Sweden) for 1 h to allow capacitation in a 37 °C incubator with 5% CO_2_. Meanwhile, oviducts were harvested from female mice, and the enlarged ampulla was punctured to allow cumulus–oocyte complexes (COCs) to be naturally released and transferred into prewarmed GIVF-PLUS medium. Next, 5 μL of capacitated sperm were added, and fertilization was carried out for 6 h in the incubator. After fertilization, zygotes were washed three times in fresh prewarmed GIVF-PLUS medium and transferred to G1-PLUS medium (Vitrolife, Gothenburg, Sweden) for further culture. The total numbers of denuded oocytes retrieved after superovulation, two-cell embryos at 24 h, four-cell embryos at 48 h, eight-cell embryos at 60 h, morula at 72 h, and blastocysts at 96 h post-fertilization, were recorded. Embryos were cultured at 37 °C under 5% O_2_ and 6% CO_2_ conditions in a MINC incubator (Cook Medical, Bloomington, IN, USA). For superovulation and IVF assays, *N* = 3–12 mice per group per time point were analyzed.

### 2.6. Evaluation of Offspring After Birth

Pups did not receive any postnatal intervention. From birth to adulthood, F1 offspring were monitored daily, with separate assessments of physical growth, neurodevelopmental, and sexual development. Upon reaching adulthood, learning ability and reproductive function were evaluated. For pre-weaning offspring assessments, the litter was used as the experimental unit for statistical analysis. In contrast, adult offspring experiments, including the Morris Water Maze test and fertility assessments, were analyzed using individual mice as the statistical unit, and mice were randomly selected for each experiment.

### 2.7. Physical Development

Physical development was evaluated based on body weight gain, bilateral pinna detachment, lower incisor eruption, and eyes opening. Body weight was measured on postnatal days 1, 4, 7, 14, 21, 28, 35 and 42. Weaning was performed on postnatal day 21, after which pups were separated from their dams and grouped by sex. From weaning onward, body weights of males and females were recorded separately. Bilateral pinna detachment was assessed between postnatal days 4 and 7, and the number of pups per litter exhibiting fully unfolded external ears was recorded. Lower incisor eruption was evaluated between postnatal days 11 and 14, defined by the emergence of incisors through the gingiva. Eyes opening was assessed between postnatal days 16 and 20, and the number of pups per litter with both eyes fully open was recorded. Body weight before weaning was evaluated using *N* = 9–13 litters per group, and the mean body weight of all pups within each litter was recorded. Bilateral pinna detachment (*N* = 8–13 litters/group), lower incisor eruption (*N* = 6–13 litters/group), and eye opening (*N* = 6–11 litters/group) were analyzed as the percentage of pups within each litter meeting the corresponding developmental criteria.

### 2.8. Neurobiological Development

Three behavioral tests were conducted to assess neurodevelopment in pups. Cliff avoidance was assessed on postnatal day 8 [31,32]. Each pup was placed on a horizontal platform approximately 20 cm above the tabletop, with its forepaws and nose extending slightly beyond the edge. A positive response was defined as movement away from the edge within 30 s, and the number of pups per litter meeting the corresponding criterion was recorded. Negative geotaxis was evaluated on postnatal day 9 [33,34]. Pups were positioned head-down on an acrylic surface inclined at a 45°, and successful performance was defined as a 180° rotation within 30 s. The number of pups per litter achieving this response was documented. The surface righting reflex was assessed on postnatal day 10 [32]. Pups were placed in a supine position on a flat surface for 4 s and then released. A successful response was defined as regaining contact with all four paws within 2 s, and the number of pups per litter fulfilling this criterion was recorded. Behavioral assessments were performed using *N* = 8–13 litters per group, and the percentage of pups within each litter fulfilling the corresponding criteria was used for statistical analysis.

### 2.9. Sexual Development

Puberty onset was evaluated based on testicular descent in male pups and vaginal opening in female pups. Testicular descent was monitored from postnatal days 22 to 29. The scrotum was palpated daily to confirm the migration of testes from the abdominal cavity into the scrotum [35]. The number of pups per litter exhibiting complete testicular descent was recorded each day. Vaginal opening was used as the marker of puberty onset in female pups [33]. Each day, the tail of each female pup was gently lifted to directly observe the vaginal opening. Vaginas were lightly swabbed with a cotton swab: closed vaginas remained attached to the surrounding skin, whereas open vaginas were separated. The number of female pups per litter with open vaginas was recorded daily. Puberty assessments included *N* = 3–13 litters per group, and the percentage of pups within each litter meeting the corresponding criteria was used for statistical analysis.

### 2.10. Morris Water Maze Test

The Morris Water Maze consisted of a circular pool divided into four quadrants, with a hidden platform placed in quadrant III. The platform was submerged 1–2 cm below the water surface, with the water made opaque by adding skimmed milk powder before each trial. The experiment was conducted over six consecutive days. Mice were allowed to acclimate to the testing environment for 1 h prior to each session. During the first five days (acquisition training phase), animals were released sequentially from each quadrant and allowed up to 60 s to locate the hidden platform. Upon reaching the platform, mice remained there for 15 s. If the platform was not located within the allotted time, mice were gently guided to it and allowed to remain for 15 s. On day 6, a probe trial (spatial exploration phase) was performed with the platform removed. Mice were released from quadrant I and allowed to swim freely for 60 s to assess spatial memory retention of the former platform location (quadrant III). Swimming trajectories were recorded using the ANY-maze tracking system (Stoelting Co., Wood Dale, IL, USA). Performance was evaluated based on the frequency of crossings over the former platform location and the percentage of time spent in the target quadrant. Morris Water Maze testing included *N* = 5–8 randomly selected adult offspring mice per group.

### 2.11. Fertility Assessment of F1 Mice

Fertility of male and female F1 mice was assessed separately. Female offspring underwent fertility evaluation using the same protocol as that applied to maternal mice. For male fertility evaluation, sperm forward motility, total sperm motility, and sperm concentration were analyzed. Briefly, the cauda epididymis was excised and placed in 1 mL of pre-warmed phosphate-buffered saline (PBS). The tissue was finely minced and incubated at 37 °C for 30 min to facilitate sperm release. Following incubation, 500 μL of the supernatant was collected, and a 10 μL aliquot was placed on a glass slide with a coverslip for microscopic examination. Using a blood cell counting chamber, at least 200 spermatozoa were counted across multiple fields and classified as forward motile, non-forward motile, or immotile. In parallel, 100 μL of the sperm suspension was mixed with 4% paraformaldehyde at a 1:1 ratio to preserve sperm for concentration analysis. Sperm concentration was subsequently determined using a cell counter (Bio-Rad, Hercules, CA, USA). Total motility was defined as the sum of the percentages of forward motile and non-forward motile sperm. Fertility evaluation of F1 female offspring included *N* = 7–8 randomly selected mice per group, while fertility evaluation of F1 male offspring included *N* = 3–6 randomly selected mice per group.

### 2.12. Statistical Analysis

Continuous data were presented as mean ± standard error of the mean (SEM) and analyzed using GraphPad Prism (version 10; GraphPad Software, San Diego, CA, USA). Normality was assessed using the Shapiro–Wilk test. For normally distributed data, homogeneity of variance was evaluated using Levene’s test. Comparisons between two independent groups were performed using the unpaired *t*-test when variances were equal, or Welch’s *t*-test when variances were unequal. Non-normally distributed data were analyzed using the Mann–Whitney U test. Categorical variables, such as the numbers of pregnant versus non-pregnant female mice, were analyzed using the chi-square test, Yates’ continuity-corrected chi-square test, or Fisher’s exact test, as appropriate. The chi-square test was applied when the total sample size (*N*) was ≥40 and all expected frequencies were ≥5. Yates’ continuity-corrected chi-square test was used when *N* ≥ 40 but at least one expected frequency was between 1 and 5. Fisher’s exact test was used when *N* < 40 or any expected frequency was <1. Results were presented as *n* (%) or percentages.

All statistical tests were two-sided, and *p* < 0.05 was considered statistically significant. Statistical significance was denoted as * *p* < 0.05 and ** *p* < 0.01.

**Figure 1:**
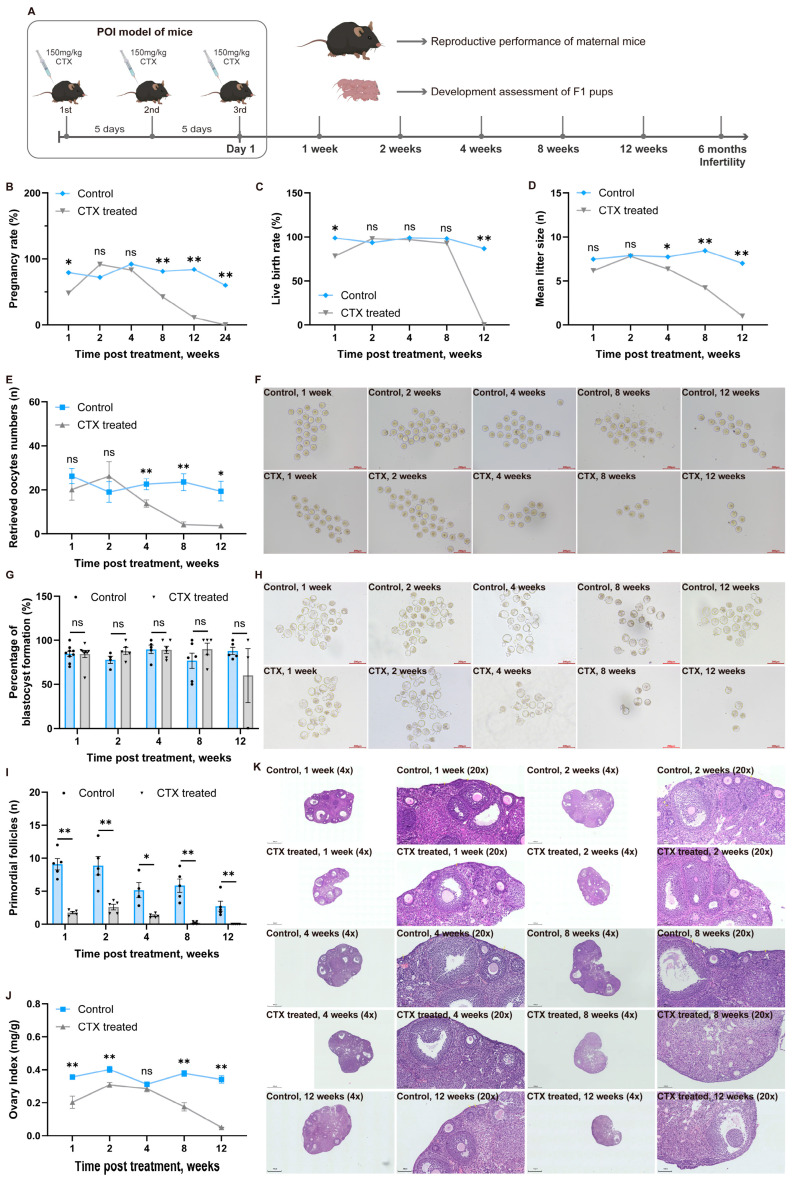
**Reproductive performance evaluated at different time intervals in maternal mice following CTX treatment.** (**A**) Schematic illustration of the experimental design. (**B**–**D**) Dynamics of fertility parameters, including (**B**) pregnancy rate, (**C**) live birth rate, and (**D**) mean litter size. (**E**) Number of total oocytes retrieved after superovulation at different time points. (**F**) Representative photomicrographs of retrieved oocytes after superovulation. Scale bar, 200 μm. (**G**) Embryonic developmental potential assessed by blastocyst formation rate (blastocysts/2-cell embryos) following IVF. (**H**) Representative photomicrographs of blastocysts after IVF. Scale bar, 200 μm. (**I**) Primordial follicle counts at various time points after repeated CTX administration. (**J**) Changes in ovary index following CTX treatment. (**K**) Representative H&E-stained ovarian sections. Scale bars, 500 μm and 100 μm. Representative primordial follicles are marked with arrows. * *p* < 0.05, ** *p* < 0.01.

**Figure 2:**
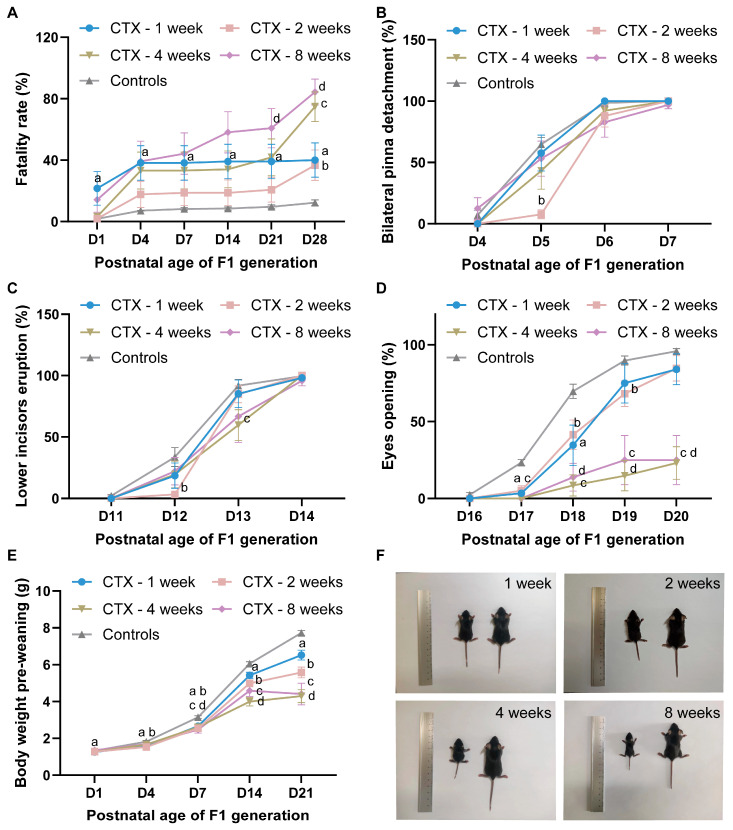
**Fatality rates and growth parameters in preweaning offspring.** (**A**) Fatality rates of offspring from different groups during the first four weeks after birth. (**B**–**E**) Physical development parameters across groups, including (**B**) bilateral pinna detachment, (**C**) lower incisor eruption, (**D**) eyes opening, and (**E**) body weight gain from birth to weaning. (**F**) Representative images of pups at weaning (postnatal day 21) from CTX-treated (left) and control (right) groups. a *p* < 0.05, control versus CTX at 1 week; b *p* < 0.05, control versus CTX at 2 weeks; c *p* < 0.05, control versus CTX at 4 weeks; d *p* < 0.05, control versus CTX at 8 weeks.

**Figure 3:**
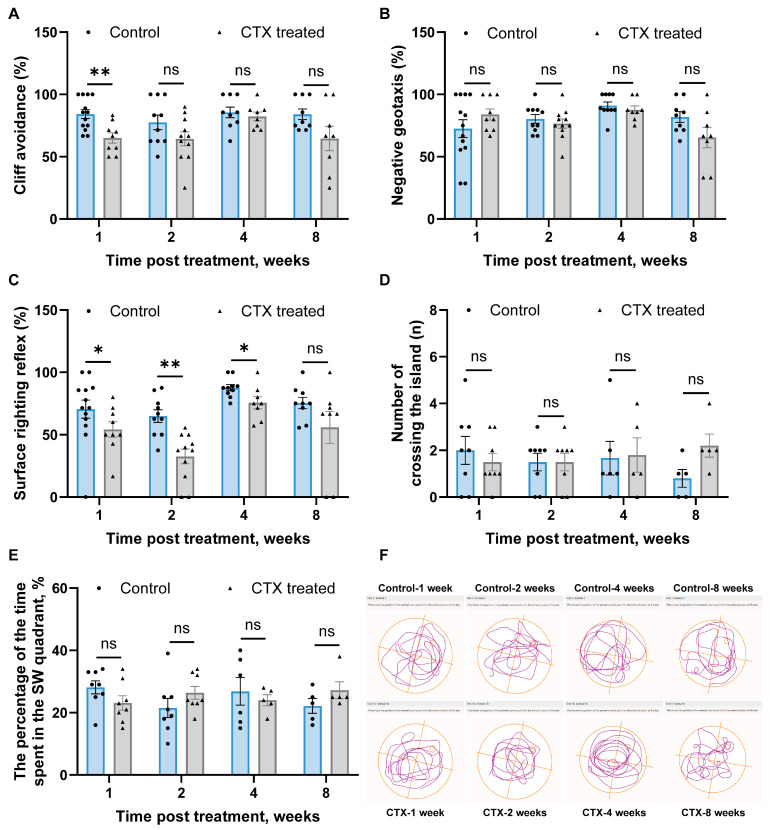
**Behavioral tests in F1 mice from preweaning to adulthood.** Behavioral assessments during preweaning included neurodevelopmental tests: (**A**) cliff avoidance, (**B**) negative geotaxis, and (**C**) surface righting reflex tests conducted on postnatal days 8, 9, and 10, respectively. Behavioral assessments during adulthood included Morris water maze test: (**D**) number of crossings over the original platform area within 60 s, (**E**) percentage of time spent in the target quadrant within 60 s, (**F**) Representative trajectory of mice in the maze within 60 s. * *p* < 0.05, ** *p* < 0.01.

**Figure 4:**
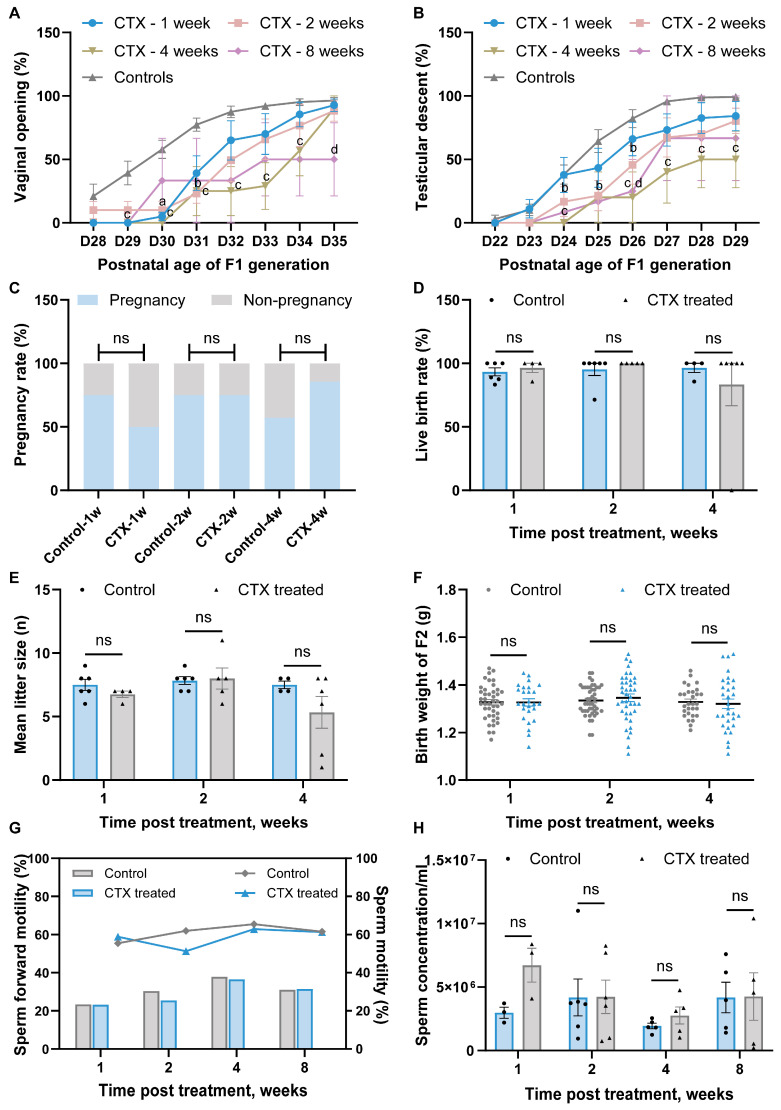
**Gonadal function in F1 mice from prepubertal to adulthood.** Puberty onset in prepubertal F1 mice: (**A**) vaginal opening in females and (**B**) testicular descent in males on postnatal days 22–29 and 28–35, respectively. Fertility parameters of female offspring included (**C**) pregnancy rate, (**D**) live birth rate, (**E**) mean litter size, and (**F**) birth weight of F2 offspring. Fertility parameters of male offspring included: (**G**) sperm forward motility (bar charts), and sperm motility (line charts), and (**H**) sperm concentration. a *p* < 0.05, control versus CTX at 1 week; b *p* < 0.05, control versus CTX at 2 weeks; c *p* < 0.05, control versus CTX at 4 weeks; d *p* < 0.05, control versus CTX at 8 weeks.

Each treatment group was matched with a corresponding time-point control group, and comparisons were performed between each group and its matched control (see Tables S1–S5). For clarity in Figure 1, Figure 2, Figure 3 and Figure 4, control groups from different time points were pooled for presentation as mean ± SEM, while statistical analyses were performed using the original time-matched controls.

## 3. Results

### 3.1. Reproductive Performance After CTX Treatment

To elucidate the effects of the CTX regimen on female fertility, we assessed pregnancy outcomes at various intervals following administration of a clinically relevant dose (150 mg/kg × 3) [6,8,14,26] (Figure 1A). One week after completing CTX treatment, fertility was significantly impaired, as evidenced by significant reductions in pregnancy rate (*p* < 0.05, Figure 1B and Table S1) and live birth rate (*p* < 0.05, Figure 1C and Table S1), accompanied by a downward trend in mean litter size (Figure 1D and Table S1). By two weeks post-administration, fertility parameters returned to levels comparable to controls (Figure 1B–D, and Table S1). However, at four weeks post-chemotherapy, mean litter size declined again (*p* < 0.05, Figure 1D and Table S1), although pregnancy rate and live birth rate remained similar to controls (Figure 1B,C and Table S1). At eight weeks, live birth rate was comparable to controls (Figure 1C), yet both pregnancy rate and mean litter size (*p* < 0.01, Figure 1B,D) were significantly reduced. By twelve weeks, pregnancy rate dropped dramatically (*p* < 0.01, Figure 1B), and no live pups were born (Figure 1C). Six months post-CTX treatment, female mice were completely infertile following natural mating (*p* < 0.01, Figure 1B and Table S1).

Ovarian stimulation can affect oocyte yield; therefore, we evaluated the number of oocytes retrieved after superovulation and the developmental competence of embryos following IVF (Figure 1E–H). One week after CTX cessation, oocyte retrieval tended to decrease, although the difference was not statistically significant (Figure 1E and Table S2). At two weeks, the number of retrieved oocytes showed an upward trend (Figure 1E and Table S2). Beginning four weeks post-treatment, oocyte yield declined sharply and continued to decrease, with almost no oocytes retrieved by twelve weeks (Figure 1E and Table S2). IVF experiments demonstrated that blastocyst formation rate was comparable to controls (Figure 1G,H and Table S2). To further evaluate the effect of CTX on ovarian reserve, follicle counts were performed (Figure 1I,K). CTX caused a significant and irreversible depletion of primordial follicles compared to controls (Figure 1I and Table S2). By twelve weeks post-treatment, primordial follicles were nearly exhausted (Figure 1I,K), and early growing follicles (primary plus secondary) and antral follicles had almost completely disappeared (Figure 1K, Table S2). Correspondingly, ovarian tissues showed severe atrophy at twelve weeks (Figure 1J,K).

### 3.2. CTX-Induced Increased Fatality and Growth Retardation in Offspring

We assessed the survival and physical development of surviving pups, with the corresponding parameters summarized in Figure 2 and Table S3. Fatality rates were higher in all CTX-treated groups (1–8 weeks) compared with controls, and fatality progressively increased with age (Figure 2A). On postnatal day 28, the highest fatality rate was observed in the 8-week CTX group, followed by the 4-week group, whereas the 2-week group exhibited the lowest fatality rate.

Physical development was assessed by monitoring bilateral pinna detachment (Figure 2B, postnatal days 4–7), lower incisor eruption (Figure 2C, postnatal days 11–14), eyes opening (Figure 2D, postnatal days 16–20), and body weight gain from birth to weaning (Figure 2E, postnatal days 1–21). During early postnatal development, pups in the CTX-treated groups exhibited delayed bilateral pinna detachment on postnatal day 5 in the 2-week group, as well as delayed lower incisor eruption on postnatal day 12 in the 2-week group and postnatal day 13 in the 4-week group. With increasing postnatal age, pups in CTX groups exhibited delayed eyes opening (Figure 2D), particularly in the 4- and 8- week groups. Differences in body weight gain became more pronounced over time. The 8-week group exhibited the greatest reduction in body weight at weaning, followed by the 4-week, 2-week, and 1-week groups (Figure 2E). Consistently, pups from all CTX-treated groups were smaller at weaning (postnatal day 21) than controls, with the most pronounced reduction observed in the 8-week group (Figure 2F).

### 3.3. CTX-Induced Neurobehavioral Deficits During Early Development but No Effect on Learning and Memory in Adult Offspring

To evaluate the neurological development of pre-weaning pups, we conducted three behavioral tests: cliff avoidance on postnatal day 8, negative geotaxis on postnatal day 9, and the surface righting reflex on postnatal day 10. Figure 3 and Table S4 provided a summary of the findings. Compared with controls, pups in all CTX-treated groups showed a decreasing trend in cliff avoidance performance at all time points, with a statistically significant reduction observed at 1 week (Figure 3A). Maternal CTX exposure had no effect on negative geotaxis in offspring (Figure 3B). Significant impairments were observed across the treated groups in the surface righting reflex test, except for the 8-week group, which showed only a decreasing trend (Figure 3C). In the offspring that survived to adulthood, learning and memory were assessed using the Morris Water Maze. Performance was evaluated by the frequency of crossings over the original platform location and the percentage of time spent in the target quadrant (Figure 3D–F). No significant differences were observed between the CTX-treated and control groups, indicating that maternal CTX exposure did not impair learning and memory in offspring that survived to adulthood.

### 3.4. CTX-Induced Delayed Onset of Puberty in Offspring but Did Not Affect Their Fertility in Adulthood

Puberty onset was assessed by monitoring vaginal opening in female pups and testicular descent in male pups. In the CTX-treated groups, female pups exhibited delayed vaginal opening during puberty, with the 8-week CTX group showing the most pronounced delay (Figure 4A). Similarly, male pups displayed delayed testicular descent, with the 4-week and 8-week CTX groups being most affected (Figure 4B). In the offspring that survived to adulthood, fertility in female offspring was evaluated by mating them with male mice, while male fertility was assessed by analyzing sperm parameters. Among adult female offspring, no significant differences were observed in pregnancy rate, live birth rate, mean litter size, or birth weight of the F2 generation between CTX-treated and control groups (Figure 4C–F, and Table S5). Due to the high lethality in the 8-week group, fewer than three female offspring survived to adulthood, precluding adequate mating experiments in this group. Similarly, male offspring showed no significant differences in sperm forward motility, total motility, or sperm concentration (Figure 4G,H, and Table S5).

## 4. Discussion

In studies of long-term survivors, clear and consistent evidence regarding pregnancy and offspring outcomes following CTX treatment remains limited, often due to insufficient details on dosage, variability in the interval between exposure and conception, and the use of combination regimens. Similarly, rodent studies have reported inconsistent findings, influenced by differences in drug exposure levels and observational parameters. Consequently, the obstetric and offspring risks associated with prior CTX exposure in female mice are inadequately explored. This study provides a comprehensive assessment of the adverse effects of CTX on maternal reproductive potential and offspring health.

We revealed the dynamic effects of high-dose CTX administration on maternal fertility. After mating with healthy males, pregnancy rate, mean litter size, live birth rate, and birth weight of F1 offspring initially declined, showed signs of recovery at 2 weeks, and then decreased again at 4 weeks, followed by progressive deterioration. By 6 months post-medication, CTX ultimately led to infertility, aligning with CTX-induced premature menopause in humans [10,19]. These findings confirm that CTX-induced depletion of ovarian reserve ultimately shortens the reproductive lifespan in mice [36,37]. To further substantiate these observations, we performed ovarian hyperstimulation assays. The number of oocytes retrieved after superovulation followed a pattern similar to that of mean litter size, reflecting the impact of CTX on follicular reserve. The strong concordance between litter size and oocyte yield supports the robustness of our reproductive outcome data.

The detrimental effects of CTX on the ovary involve both acute and chronic mechanisms. CTX preferentially targets mitotically active somatic cells, such as granulosa cells [38], leading to the destruction of growing follicles through the induction of granulosa cell apoptosis [39]. CTX can also directly damage oocytes [39,40]. This aligns with our observation of reduced mean litter size at 1 week post-chemotherapy, indicating the acute destructive effect of CTX on late pre-antral follicles immediately following chemotherapy [28,41]. At 2 weeks after the final administration, which marks 3 weeks from the initial dose (the duration required for primordial follicles to develop into ovulatory follicles in mice [39,42]), the transient restoration of fertility may be attributed to CTX-induced activation of primordial follicles [43,44]. Loss of growing follicles disrupts the negative regulatory mechanisms that restrain primordial follicle activation, thereby triggering accelerated recruitment and development [39]. With longer discontinuation intervals, the long-term effects of CTX gradually become evident, likely due to both direct and indirect ovarian damage mediated by CTX. Firstly, CTX can directly cause DNA damage and apoptosis in primordial follicles [40]. Consequently, outcomes observed four weeks after drug withdrawal likely reflect damage to primordial follicles previously exposed to CTX. Furthermore, CTX disrupts the ovarian microenvironment by decreasing blood vessel density [45] and promoting ovarian fibrosis [46,47], potentially impairing oocyte development in the long term and ultimately reducing fertility. During this process, the limited pool of remaining primordial follicles develops within a progressively deteriorated ovarian microenvironment, adversely affecting both their quantity and quality, thereby contributing to adverse reproductive and offspring outcomes. Therefore, maternal reproductive impairment became progressively more severe in mice with longer intervals between the last CTX administration and mating. Consistent with this hypothesis, fertility outcomes and oocyte yield declined beginning at 4 weeks after CTX administration, deteriorating further thereafter. By 12 weeks, CTX-treated ovaries exhibited severe atrophy. Although a small number of early- and late-stage growing follicles were still detectable, the numbers of retrieved oocytes, pregnancy rate, and mean litter size were extremely low, with no live births. By 6 months post-CTX administration, complete infertility was observed, which is consistent with the near exhaustion of primordial follicles already evident at 12 weeks. Collectively, our findings indicate that, in the absence of protective interventions, CTX-induced fertility damage is progressive and irreversible, even after prolonged drug discontinuation.

We found that the blastocyst formation rate of embryos derived from IVF was not significantly affected by prior maternal CTX exposure, consistent with previous studies [48]. However, this finding appears inconsistent with the reduced live birth rate observed in vivo. One possible explanation is that follicles exposed to CTX may sustain DNA damage while failing to undergo complete apoptosis. Previous research has shown that DNA damage in oocytes does not necessarily lead to apoptosis. Specifically, 12 h after CTX exposure, 94% of oocytes exhibited DNA damage, whereas only 22% underwent apoptosis at 24 h [40]. This means that DNA-damaged oocytes may continue to mature, undergo fertilization, and form blastocysts, yet carry sublethal genomic or epigenetic abnormalities. Although such embryos may reach the blastocyst stage or even result in apparently normal neonates, underlying defects could compromise implantation success, fetal development, or long-term offspring health. Pregnancy outcomes are influenced not only by embryonic developmental competence but also by the maternal reproductive environment in vivo. Therefore, although IVF parameters appeared relatively normal, the in vivo reproductive outcomes did not fully align with these findings. An alternative explanation is that chemotherapy induces off-target damage to the maternal uterus, vasculature, and/or placental environment, thereby contributing to adverse pregnancy outcomes [3].

Our results revealed that maternal CTX treatment impacted multiple domains of postnatal development in the F1 generation, including physical, neurodevelopment, pubertal issues, as well as survival. However, the mice that survived into adulthood exhibited learning ability and fertility comparable to controls. Consistent with the progressive impairment in maternal reproductive function, increased lethality in F1 offspring also became more pronounced with longer intervals between the last CTX administration and mating, with the highest fatality observed in the 4- and 8-week groups. This may reflect increased vulnerability of F1 offspring derived from primordial follicles previously exposed to CTX compared with those derived from growing follicles. One possible explanation is that the off-target and long-term damage of CTX on the ovary and uterus may adversely affect oocyte quality and the intrauterine environment through fibrosis, endometrial injury, or impaired vascularization. Under these conditions, offspring derived after longer withdrawal intervals may exhibit more severe developmental abnormalities and higher mortality risk. In addition, some latent developmental defects may progressively manifest and worsen during postnatal development, thereby further increasing offspring fragility and lethality.

Given that weight gain is a critical indicator of growth and closely associated with developmental milestones [49], the reduced weight gain observed in the CTX group suggests broader impairments in physical development, including delayed pinna detachment, delayed lower incisor eruption, and delayed eyes opening. Fascinatingly, offspring of the same age exhibited considerable variability in body size, with those from the 4-week and 8-week CTX groups showing marked growth retardation (Figure 2F).

Cliff avoidance, negative geotaxis, and the surface righting reflex are commonly used behavioral tests in developmental neurotoxicity assessment. The cliff avoidance test assesses cognitive function and behavioral abnormalities by evaluating the integration of exteroceptive input and locomotor output, which may be influenced by motor, arousal, or cognitive dysfunction [50]. Our results showed that offspring from CTX groups had a lower tendency to avoid danger, suggesting that maternal CTX exposure impairs cognitive function, leading to reduced ability to recognize and avoid elevated places. The negative geotaxis assay, an automatic response to geogravitational stimuli, requires intact vestibular input to sense body orientation on an inclined plane and coordinated motor activity mediated by the cerebellum [51]. No significant abnormalities were observed in the CTX groups, suggesting that CTX exposure did not impair the maturation of central nervous system structures involved in motor coordination, particularly the cerebellum. The surface righting reflex is a test of labyrinth function and postural control, and delays in its development may indicate impaired neuronal myelination [52]. Pups in the CTX group performed worse on this test, suggesting that maternal CTX exposure may impair neuronal myelination. These findings suggest that the observed phenotype may not be fully explained by generalized growth delay alone and raise the possibility that maternal CTX exposure may additionally affect specific aspects of neurodevelopment.

The mechanisms underlying the adverse effects of maternal CTX exposure on offspring remain unclear, and available evidence is insufficient to fully explain these findings. Nevertheless, several potential explanations may be considered. First, neuronal development in the offspring may have been impaired, affecting neuronal function. One study reported reduced levels of ELK1, a factor involved in neuroprotection against toxic stimuli, in the whole brain, and decreased AKT1 expression, which is associated with neuronal development and overall growth, in the frontal cortex of offspring from paternal CTX exposure [53]. Additionally, offspring of CTX-exposed fathers exhibited increased postnatal mortality, impaired learning ability, and reduced spontaneous activity [54]. These findings suggest that CTX may induce biochemical alterations in the brain of progeny. Second, nutritional status may have contributed to the delayed behavioral responses observed in pups. Impaired intrauterine growth or insufficient nutritional support may compromise the development of sensory organs and the neuromuscular system, thereby affecting sensory perception and muscle strength. Moreover, cognitive factors may further modulate behavioral performance. Overall, interpretation of behavioral outcomes in isolation remains challenging due to the multifactorial nature of neurodevelopmental processes.

Although CTX exposure affected nervous system maturation in pre-weaning pups, subsequent assessments demonstrated that adult offspring in the CTX group exhibited normal learning and memory capacities. This finding is consistent with another study reporting no cognitive differences between adult offspring of the CTX-treated and control groups, as evaluated by the Y-maze test and novel object recognition task [55]. Despite the increased postnatal mortality observed in CTX-exposed groups, surviving offspring that reached adulthood exhibited normal fertility. However, because prenatal loss and the specific causes of neonatal death were not systematically evaluated in the current study, the relationship between early developmental abnormalities, mortality, and long-term functional outcomes remains unclear and warrants further investigation.

### Limitations and Implications for Future Research

This study primarily focused on longitudinal phenotypic outcomes following maternal CTX exposure and did not investigate the molecular and regulatory mechanisms underlying the observed developmental abnormalities in offspring. Future studies incorporating transcriptomic, epigenetic, and whole-genome analyses may help elucidate the molecular basis of these abnormalities and facilitate the potential application of preimplantation genetic testing (PGT) for early detection of developmental risks and prediction of pregnancy outcomes in cancer survivors. Additionally, we did not directly assess oocyte quality or comprehensively evaluate the potential adverse effects of CTX on the uterus and intrauterine environment. Embryonic resorption rates were also not systematically assessed by uterine examination at defined gestational stages. Although pregnancy rate, litter size, live birth rate, and stillbirth events were recorded, these parameters provide only indirect information regarding prenatal loss. Future studies incorporating timed uterine examination during gestation may help differentiate implantation failure, embryonic resorption, fetal malformations, and postnatal mortality following maternal CTX exposure. Furthermore, histopathological analyses of key organs in F1 offspring, including the brain, ovary, testis, liver, and heart, were not performed in the current study. Such analyses would provide important structural and mechanistic insights into the observed developmental abnormalities and may help distinguish generalized developmental delay from organ-specific toxicity. Future studies incorporating histopathological, molecular, and mechanistic analyses will be necessary to further characterize the long-term effects of maternal CTX exposure on reproductive health and offspring development.

## 5. Conclusions

In conclusion, maternal CTX treatment induces stage-specific reproductive damage, beginning with acute injury, followed by partial recovery, chronic damage, and subsequent deterioration, ultimately resulting in infertility. CTX-induced damage in F1 offspring persists and shows no evidence of recovery following prolonged drug withdrawal. Despite a higher likelihood of pregnancy at 2 weeks after cessation, with comparatively favorable pregnancy and obstetric outcomes, caution is warranted due to associated risks of delayed physical development, behavioral abnormalities, increased postnatal fatality, and delayed pubertal onset in offspring. Notably, more severe adverse outcomes were observed at 4 and 8 weeks, indicating that extending the withdrawal interval did not further improve offspring outcomes. Therefore, cancer survivors with reproductive goals should seek pre-conception counseling early and consider fertility preservation strategies, such as ovarian tissue, embryo cryopreservation, or oocyte cryopreservation prior to chemotherapy, to mitigate these risks.

## Data Availability

The data underlying this article are available in the article and in its online Supplementary Materials.

## Supplementary Materials

The following supporting information can be downloaded at: https://www.mdpi.com/article/10.3390/biomedicines14061353/s1, Table S1: Fertility outcomes in F0 mice after mating with healthy males. Table S2: Follicles count, oocyte number, embryo development in F0 mice. Table S3: Physical developmental characteristics and mortality rate in F1 mice. Table S4: Behavioral test characteristics in F1 mice preweaning and postweaning. Table S5: Reproductive system performance during puberty and adulthood in F1 mice. Figure S1: Representative histological images and classification of ovarian follicles.

## Abbreviations

CTX	Cyclophosphamide
COC	cumulus-oocyte complex
hCG	Human Chorionic Gonadotropin
H&E	hematoxylin and eosin
IVF	in vitro fertilization
PBS	phosphate-buffered saline
PGT	preimplantation genetic testing
PMSG	Pregnant Mare Serum Gonadotropin
SEM	standard error of the mean
SPF	specific pathogen-free
