# Supplementary material for: From Maternal Exposure to F1 Development: Unveiling Cyclophosphamide-Induced Reproductive Toxicity
Source: Biomedicines. 2026 Jun 16;14(6):1353. doi: 10.3390/biomedicines14061353 (PMC13297049; doi:10.3390/biomedicines14061353)
Supplement: Supplementary file 1 [file biomedicines-14-01353-s001.zip › biomedicines-4291845-supplementary.pdf]

Supplement

Table S1 Fertility outcomes in F0 mice after mating with healthy males. 2

Table S2 Follicles count, oocyte number, embryo development in F0 mice. 3

Table S3 Physical developmental characteristics and mortality rate in F1 mice. 4

Table S4 Behavioral test characteristics in F1 mice preweaning and postweaning. 7

Table S5 Reproductive system performance during puberty and adulthood in F1 mice ... 8

Figure S1 Representative histological images and classification of ovarian follicles. 11

Table S1 Fertility outcomes in F0 mice after mating with healthy males.

Parameters	1 week post treatment		2 weeks post treatment		4 weeks post treatment		8 weeks post treatment		12 weeks post treatment		6 months post treatment	
	**Control**	**CTX**	**Control**	**CTX**	**Control**	**CTX**	**Control**	**CTX**	**Control**	**CTX**	**Control**	**CTX**
**F0 mice numbers, N**	24	25	25	24	13	18	32	33	25	27	15	15
**Pregnancy rate, N (%)**												
Pregnancy	19 (79.167%)	12 (48%)	18 (72%)	22 (91.667%)	12 (92.308%)	15 (83.333%)	26 (81.250%)	14 (42.424%)	21 (84%)	3 (11.111%)	9 (60%)	0 (0%)
Non-pregnancy	5 (20.833%)	13 (52)[a]	7 (28%)	2 (8.333%)	1 (7.692%)	3 (16.667%)	6 (18.750%)	19 (57.576%)[b]	4 (16%)	24 (88.889%)[b]	6 (40%)	15 (100%)[b]
**Mean litter size, Mean±SEM**	7.474±0.362	6.167±0.806	7.882±0.241	7.81±0.335	7.727±0.384	6.357±0.498[a]	8.450±0.456	4.214±0.526[b]	7±0.488	1±0[b]		
**Live birth rate, %, Mean±SEM**	98.76±0.856	78.32±10.95[a]	93.58±3.545	97.91±0.969	99.09±0.909	96.79±2.199	98.13±1.368	92.86±4.853	86.85±10.96	0±0[b]		

[a]$P < 0.05$, [b]$P < 0.01$.

Table S2 Follicles count, oocyte number, embryo development in F0 mice.

Parameters	1 week post treatment		2 weeks post treatment		4 weeks post treatment		8 weeks post treatment		12 weeks post treatment	
	Control	CTX	Control	CTX	Control	CTX	Control	CTX	Control	CTX
**Follicle counts per section**										
Primordial follicles, Mean±SEM (N)	9.08±0.866 (N=5)	1.74±0.178[b] (N=5)	8.86±1.394 (N=5)	2.6±0.42[b] (N=5)	5.15±1.147 (N=4)	1.26±0.133[a] (N=5)	5.84±1.007 (N=5)	0.2±0.089[b] (N=5)	2.72±0.747 (N=5)	0±0[b] (N=5)
Early growing follicles, Mean±SEM (N)	11.32±0.595 (N=5)	5.84±1.513[b] (N=5)	9.8±1.792 (N=5)	6.28±0.761 (N=5)	10.3±2.431 (N=4)	4.94±0.855 (N=5)	6.92±1.179 (N=5)	3±0.699[a] (N=5)	6.84±1.065 (N=5)	2.68±1.248[a] (N=5)
Antral follicles, Mean±SEM (N)	5.68±1.177 (N=5)	2.7±0.902 (N=5)	4.54±0.166 (N=5)	2.94±0.275[a] (N=5)	3.25±0.568 (N=4)	1.68±0.35[a] (N=5)	2.36±0.366 (N=5)	0.64±0.194[b] (N=5)	2.6±0.438 (N=5)	0.16±0.117[b] (N=5)
**Retrieved oocytes numbers (N)**	26.222±3.431 (N=9)	20.125±4.849 (N=8)	19±4.673 (N=4)	26.2±6.621 (N=5)	22.556±2.478 (N=9)	13.75±1.624[b] (N=12)	23.5±3.822 (N=8)	4.2±1.241[b] (N=5)	19.4±4.468 (N=5)	3.667±0.882[a](N=3)
**IVF**										
Percentage of 2 cell formation (%)	91.08±1.804 (N=9)	93.994±2.774 (N=8)	90.921±3.263(N=4)	86.601±3.499 (N=5)	90.309±4.323 (N=5)	92.222±4.12 (N=6)	75.598±9.272 (N=6)	91.667±5.27 (N=5)	79.227±3.761 (N=5)	83.333±16.667 (N=3)
Percentage of blastocyst formation (%)	84.698±3.025 (N=9)	84.442±4.09 (N=8)	77.732±4.035 (N=4)	87.79±4.092 (N=5)	89.67±4.734 (N=5)	89.222±3.977 (N=6)	76.802±8.579 (N=6)	90±6.667 (N=5)	87.9±4.15 (N=5)	60±30.551 (N=3)

Values expressed as Mean ± SEM, [a]$P < 0.05$, [b]$P < 0.01$.

Table S3 Physical developmental characteristics and mortality rate in F1 mice.

Parameters	1 week post treatment		2 weeks post treatment		4 weeks post treatment		8 weeks post treatment	
	Control	CTX	Control	CTX	Control	CTX	Control	CTX
**Body weight pre-weaning, g**								
Number of litters (D1), N	13	9	10	11	10	10	9	9
D1	1.329±0.022	1.259±0.024[a]	1.281±0.018	1.28±0.016	1.332±0.02	1.287±0.023	1.387±0.035	1.355±0.035
D4	1.723±0.047	1.574±0.047[a]	1.728±0.044	1.528±0.034[b]	1.848±0.103	1.66±0.069	2±0.174	1.688±0.107
D7	3.056±0.073	2.659±0.091[b]	2.983±0.074	2.546±0.064[b]	3.246±0.157	2.611±0.12[b]	3.314±0.268	2.475±0.191[a]
D14	5.949±0.154	5.421±0.169[a]	6.107±0.154	4.987±0.134[b]	5.837±0.134	3.993±0.231[b]	6.329±0.436	4.591±0.313[a]
D21	7.488±0.186	6.519±0.265[b]	7.551±0.177	5.58±0.294[b]	7.932±0.369	4.295±0.348[b]	7.946±0.551	4.411±0.587[b]
**Bilateral pinna detachment, %**								
Number of litters (D4), N	13	9	10	11	10	8	9	8
D4	0±0	0±0	0±0	0±0	15.143±9.175	0±0	12.963±11.034	12.5±8.768
D5	67.384±12.043	57.738±14.613	48.809±9.538	7.702±3.55[b]	75.028±11.372	43.214±15.084	68.253±13.132	53.125±14.256
D6	100±0	100±0	94.167±3.469	87.955±9.025	100±0	92.188±5.249	100±0	82.813±12.261
D7	100±0	100±0	100±0	100±0	100±0	100±0	100±0	96.875±3.125

**Lower incisors eruption, %**								
Number of litters (D11), N	13	9	10	11	9	8	9	6
D11	1.099±1.099	0±0	0±0	0±0	3.175±3.175	0±0	4.63±3.14	0±0
D12	28.801±9.705	18.624±10.359	14.346±5.238	3.283±2.393[a]	48.134±13.133	19.51±10.872	43.342±13.054	22.221±11.111
D13	98.291±1.709	85.186±11.264	77.5±13.15	84.74±6.809	91.667±8.333	59.687±12.529[a]	100±0	66.667±21.082
D14	98.291±1.709	98.148±1.852	100±0	100±0	100±0	100±0	100±0	95.833±4.167
**Eyes opening. %**								
Number of litters (D16), N	10	9	10	11	9	8	9	6
D16	0±0	0±0	0±0	0±0	5.358±5.358	0±0	4.63±3.14	0±0
D17	22.898±6.429	3.44±2.284[a]	28.036±9.792	5.176±3.156	23.894±10.381	0±0[a2]	18.827±9.257	0±0
D18	78.841±6.3	34.694±13.103[b]	74.881±9.546	41.479±9.597[a]	67.117±9.373	8.532±6.927[b]	58.068±11.868	13.888±9.044[a]
D19	96.21±1.944	75±12.817	92.917±3.931	68.156±8.305[a]	86.805±7.626	14.881±9.871[b]	83.267±7.743	25±15.957[b]
D20	100±0	84.074±10.042	94.167±3.469	84.686±8.231	96.875±3.125	23.135±10.538[b]	92.394±4.358	25±15.957[b]
**Fatality rate, %**								
D1	0.855±0.855	21.677±10.952[a]	1.429±1.429	1.968±1.329	1±1	3.214±2.199	4.167±2.946	14.286±8.168
D4	7.723±2.835	38.237±11.254[b]	7.262±3.196	17.755±8.482	6.635±3.122	33.234±12.031	6.944±3.675	39.286±13.028
D7	7.723±2.835	38.237±11.254[b]	10.595±5.692	18.796±8.347	6.635±3.122	33.234±12.031	8.179±3.589	44.286±13.454

D14	7.723±2.835	39.163±11.172[b]	11.706±6.689	18.796±8.347	6.635±3.122	34.028±11.945	8.179±3.589	58.163±13.433
D21	9.783±3.057	39.163±11.172[a]	12.956±6.561	20.764±8.045	6.635±3.122	41.851±11.972	9.414±3.447	60.842±12.793[a]
D28	9.783±3.057	40.089±11.166[a]	12.956±6.561	36.759±9.908[a]	9.313±3.001	74.83±9.66[b]	17.284±6.085	84.354±8.389[b]

Values expressed as Mean ± SEM, [a]$P < 0.05$, [b]$P < 0.01$.

Table S4 Behavioral test characteristics in F1 mice preweaning and postweaning.

	**1 week post treatment**		**2 weeks post treatment**		**4 weeks post treatment**		**8 weeks post treatment**	
**Parameters**	**Control**	**CTX**	**Control**	**CTX**	**Control**	**CTX**	**Control**	**CTX**
**Behavioral preweaning tests, %**								
Cliff avoidance (D8)	84.237±3.571 (N=13)	65.079±4.089[b] (N=9)	77.559±5.826 (N=10)	64.362±5.562 (N=11)	85.559±4.305 (N=10)	82.322±3.469 (N=8)	84.038±4.229 (N=9)	64.583±9.803 (N=8)
Negative geotaxis (D9)	72.542±7.089 (N=13)	83.916±4.44 (N=9)	80.298±3.463 (N=10)	76.558±3.741 (N=11)	91.032±2.907 (N=10)	87.678±3.08 (N=8)	81.879±4.324 (N=9)	65.401±8.205 (N=8)
Surface righting reflex (D10)	70.452±7.248 (N=13)	54.181±6.171[a] (N=9)	64.881±5.053 (N=10)	32.605±5.828[b] (N=11)	87.682±2.47 (N=10)	75.536±4.854[a] (N=8)	75.352±4.591 (N=9)	55.804±12.781 (N=8)
**Morris water maze postweaning tests**								
Number of crossing the island, n	2±0.598 (N=8)	1.5±0.378 (N=8)	1.5±0.378 (N=8)	1.5±0.378 (N=8)	1.667±0.715 (N=6)	1.8±0.735 (N=5)	0.8±0.374 (N=5)	2.2±0.49 (N=5)
The percentage of the time spent in the SW quadrant, %	28.125±2.091 (N=8)	23.125±2.31 (N=8)	21.5±3.059 (N=8)	26.375±2.061 (N=8)	26.833±4.423 (N=6)	24±1.761 (N=5)	22.2±2.354 (N=5)	27.2±2.728 (N=5)

Values expressed as Mean ± SEM, $^{a}P < 0.05$, $^{b}P < 0.01$.

Table S5 Reproductive system performance during puberty and adulthood in F1 mice

	1 week post treatment		2 weeks post treatment		4 weeks post treatment		8 weeks post treatment	
**Parameters**	**Control**	**CTX**	**Control**	**CTX**	**Control**	**CTX**	**Control**	**CTX**
**Onset of puberty**								
**Vaginal opening, %**								
Number of litters (D28), N	13	8	6	6	8	5	9	3
D28	3.846±3.846	0±0	44.444±18.593	10±6.831	8.125±5.505	0±0	27.778±12.423	0±0
D29	14.102±6.215	0±0	48.611±16.999	10±6.831	37.188±9.63	0±0[a]	57.407±15.494	0±0
D30	38.077±9.882	5±3.273[a]	56.944±16.725	10±6.831	67.291±7.513	0±0[b]	69.444±13.029	33.333±33.333
D31	71.923±10.106	39.166±13.653	68.055±12.251	23.056±7.631[a]	91.666±8.334	25±19.365[b]	77.778±12.108	33.333±33.333
D32	83.205±8.649	65±15.469	79.166±10.919	49.444±17.006	100±0	25±19.365[b]	87.5±12.5	33.333±33.333
D33	91.795±5.757	70±16.036	88.888±11.112	65.833±15.938	100±0	29±18.466[b]	87.5±12.5	50±28.868
D34	98.462±1.538	85.416±9.676	89.055±11.146	76.667±16.667	100±0	57±19.975[a]	93.333±6.667	50±28.868
D35	98.462±1.538	92.709±4.838	88.888±11.112	88.333±8.333	100±0	90±10	97.778±2.222	50±28.868[a]
**Testicular descent, %**								
Number of litters (D22), N	13	9	9	9	10	5	9	3

D22	0±0	0±0	0±0	0±0	12±9.978	0±0	0±0	0±0
D23	2.821±1.919	11.111±7.349	5.556±3.928	0±0	23±10.088	0±0	9.259±6.28	0±0
D24	18.334±6.138	37.778±13.822	50.687±8.59	16.667±11.785^{a}	49±13.008	0±0^{a}	34.074±14.717	8.333±8.333
D25	43.462±9.132	43.333±15.456	87.195±5.914	21.429±11.905^{b}	65.666±11.524	20±20	61.481±11.839	16.667±16.667
D26	64.487±7.724	66.032±13.103	96.825±3.175	45.634±15.243^{b}	78.166±9.631	20±20^{a}	89.074±5.605	25±25^{a}
D27	93.846±4.166	73.174±12.652	98.413±1.587	67.301±15.357	92.5±5.336	40±24.495^{a}	97.778±2.222	66.667±33.333
D28	95.385±3.323	82.54±12.088	100±0	70.079±14.03	100±0	50±22.361^{a}	100±0	66.667±33.333
D29	96.923±2.083	84.127±11.529	100±0	80.37±9.914	100±0	50±22.361^{a}	100±0	66.667±33.333
**Pregnancy rate, N (%)**								
Number of female F1 offspring, N	8	8	8	8	7	7		
Pregnancy	6 (75%)	4 (50%)	6 (75%)	6 (75%)	4 (57.143%)	6 (85.714%)		
Non-pregnancy	2 (25%)	4 (50%)	2 (25%)	2 (25%)	3 (42.857%)	1 (14.286%)		
Mean litter size, n), Mean±SEM	7.5±0.428	6.75±0.25	7.833±0.307	8±0.837	7.5±0.289	5.333±1.256		
Live birth rate, %, Mean±SEM	93.29±3.094	96.43±3.571	95.24±4.762	100±0	96.43±3.571	83.33±16.67		
Birth weight of F1, g, Mean±SEM	1.327±0.011	1.326±0.015	1.334±0.009	1.346±0.016	1.328±0.012	1.32±0.02		
**Sperm**								
Number of male F1 offspring, N	3	3	6	6	5	5	5	5

Sperm forward motility, %	23.35±3.345	23.21±4.378	30.41±2.446	25.42±3.584	37.88±2.83	36.5±2.155	31.02±4.515	31.47±1.893
Sperm motility, %	55.49±3.596	58.85±3.101	61.95±2.262	51.26±4.523	65.44±3.546	62.9±3.225	61.58±2.105	61.32±2.411
Sperm concentration, 10^6/ml	2.983±0.439	6.723±1.331	4.179±1.446	4.233±1.308	1.938±0.222	2.758±0.668	4.182±1.203	4.256±1.869

Values expressed as Mean ± SEM, $^{a}P < 0.05$, $^{b}P < 0.01$.

Figure S1 Representative histological images and classification of ovarian follicles.

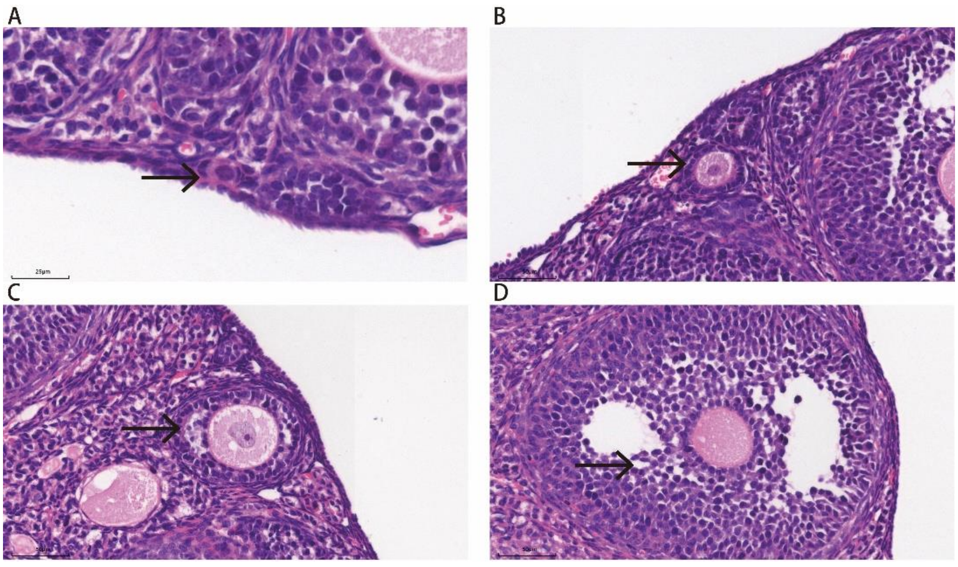

(A-D) Schematic representation of mouse ovarian follicles at different developmental stages: (A) primordial follicles (PMF), (B) primary follicles (PF), (C) secondary follicles (SF), and (D) antral follicles (AF). PMF: oocyte surrounded by a single layer of flattened granulosa cells; PF: oocyte surrounded by a single layer of cuboidal granulosa cells; SF: oocyte surrounded by multiple layers of granulosa cells without a visible antrum; AF: characterized by a clearly defined antrum.
